# A Comparative Analysis of the Ubiquitination Kinetics of Multiple Degrons to Identify an Ideal Targeting Sequence for a Proteasome Reporter

**DOI:** 10.1371/journal.pone.0078082

**Published:** 2013-10-29

**Authors:** Adam T. Melvin, Gregery S. Woss, Jessica H. Park, Lukas D. Dumberger, Marcey L. Waters, Nancy L. Allbritton

**Affiliations:** 1 Department of Chemistry, University of North Carolina, Chapel Hill, North Carolina, United States of America; 2 Department of Biomedical Engineering, University of North Carolina, Chapel Hill, North Carolina, United States of America, and North Carolina State University, Raleigh, North Carolina, United States of America; University of California, Irvine, United States of America

## Abstract

The ubiquitin proteasome system (UPS) is the primary pathway responsible for the recognition and degradation of misfolded, damaged, or tightly regulated proteins. The conjugation of a polyubiquitin chain, or polyubiquitination, to a target protein requires an increasingly diverse cascade of enzymes culminating with the E3 ubiquitin ligases. Protein recognition by an E3 ligase occurs through a specific sequence of amino acids, termed a degradation sequence or degron. Recently, degrons have been incorporated into novel reporters to monitor proteasome activity; however only a limited few degrons have successfully been incorporated into such reporters. The goal of this work was to evaluate the ubiquitination kinetics of a small library of portable degrons that could eventually be incorporated into novel single cell reporters to assess proteasome activity. After an intensive literary search, eight degrons were identified from proteins recognized by a variety of E3 ubiquitin ligases and incorporated into a four component degron-based substrate to comparatively calculate ubiquitination kinetics. The mechanism of placement of multiple ubiquitins on the different degron-based substrates was assessed by comparing the data to computational models incorporating first order reaction kinetics using either multi-monoubiquitination or polyubiquitination of the degron-based substrates. A subset of three degrons was further characterized to determine the importance of the location and proximity of the ubiquitination site lysine with respect to the degron. Ultimately, this work identified three candidate portable degrons that exhibit a higher rate of ubiquitination compared to peptidase-dependent degradation, a desired trait for a proteasomal targeting motif.

## Introduction

The ubiquitin proteasome system (UPS) is the primary pathway responsible for the recognition and degradation of misfolded, damaged, or tightly regulated proteins in addition to performing upstream roles in the signaling pathways governing DNA repair, cell cycle regulation, cell migration, and the immune response [1]. Posttranslational protein modification by ubiquitin requires a cascade of three increasingly diverse enzymes: an E1 ubiquitin activating enzyme, an E2 ubiquitin conjugating enzyme, and an E3 ubiquitin ligase. Protein ubiquitination starts with an E1 enzyme forming a high energy thioester bond with free ubiquitin, which is recognized and transferred to an E2 enzyme. Next, an E3 ubiquitin ligase forms a complex with the E2 enzyme to mediate the transfer of ubiquitin to the target protein. The E3 recognizes its target protein by binding to a specific amino acid degradation sequence, or degron. These degrons, normally in close proximity to a ubiquitin-accepting lysine residue, impart specificity to protein degradation since each E3 binds to a subset of degrons. The large number of E3 ligases (>600 in humans) permits recognition of a wide variety of degrons including phospho-degrons, oxygen dependent degrons, and N-terminal degrons [2]. The manner in which ubiquitin is transferred to a protein can occur either directly from E2 to the target protein, as is the case with RING family (Really Interesting New Gene) E3 ligases, or through an E3 ligase-bound intermediate, as is the case with HECT family (Homologous to the E6AP Carboxyl Terminus) E3 ligases [3]. Following initial ubiquitin-protein conjugation, additional ubiquitin subunits are added via one of seven different lysine residues found on ubiquitin (e.g. K48, K63, or K11) to form a polyubiquitin chain or through the N-terminal methionine residue to form a linear ubiquitin chain [4]. The residue to which the polyubiquitin chain is linked determines the fate of the conjugated protein where K48-linked chains are targeted for proteasomal degradation and K63-linked chains play a role in regulating cell signaling and DNA damage repair [1]. A polyubiquitinated protein targeted for degradation is recognized by the 19S cap of the 26S proteasome, where the target protein is deubiquitinated, unfolded, and degraded by the 20 s core particle [5]. Further, a single ubiquitin can be conjugated to the target protein, termed mono-ubiquitination, or multiple individual ubiquitins can be conjugated to multiple lysine residues near the degron, termed multi mono-ubiquitination. These differences in the number and location of protein ubiquitination significantly impact the fate of the target protein. Control of polyubiquitin chain formation is further mediated by another class of protein, deubiquitinating enzymes (DUBs), which are capable of cleaving the isopeptide bond between ubiquitin and the target protein.

Recently, dysregulation of this highly complex signaling network has been linked to multiple human diseases including cancer and neurodegenerative disorders. Proteasome inhibition by Bortezomib, and the second generation drug Carfilzomib, have shown remarkable clinical success in the treatment of patients suffering from multiple myeloma [6], [7]. While there has been a significant increase in our understanding of this complex pathway in recent years, many of the studies solely focus on the discovery of new enzymes, chaperones, or protein targets involved in the UPS. However, due to the central role that E3 ligase and proteasome dysregulation plays in a variety of cancers, it is also imperative to develop tools to not only quantify enzymatic activity, but also harness the UPS to ensure or prevent protein degradation. Recent work has demonstrated that incorporating degradation signals into native proteins results in highly effective proteasomal degradation [8], [9], while altering the initiation region of the two component degron can prevent proteasome-mediated degradation [10]. There have also been advances in assays to assess proteasomal inhibition [11], FRET-based sensors to quantify single cell proteasome activity [12], and substrates to identify the importance of protein folding on proteasomal targeting and degradation kinetics [13]. Further, tools exist that can distinguish between different ubiquitin species (free, mono-, or polyubiquitin chains) in the cytosol [14] and perform biochemical and biophysical characterization of E3 ligase kinetics independent of DUBs in a synthetic ubiquitination cascade in bacteria [15]. Additionally, novel proteomics-based approaches have been developed using mass spectrometry and stable isotope labeling by amino acids in cell culture (SILAC) to perform high throughput identification of E3 ligase substrates [16], [17]. While these reporting tools are useful, they are all limited in the analysis of patient samples due to the necessity for large sample sizes and requirement for genetic engineering of cells. To truly gain an understanding of the proteasome, reporters need to be developed to assess enzymatic activity in single primary cells. These single cell reporters can then be incorporated in highly quantitative techniques, such as capillary electrophoresis, to measure proteasome activity in cancer cells and evaluate the effectiveness of cancer therapeutics [18]–[20].

The goal of this work was to evaluate the ubiquitination kinetics of a small library of portable degrons that could eventually be incorporated into novel single cell reporters to assess proteasome activity. Currently there exists a limited number of portable degrons that have demonstrated success in targeting a reporter to the proteasome [21]; however the rate of ubiquitination of these degrons has not been fully studied or compared to other degrons. In an effort to create a proteasome reporter with the most rapid ubiquitination kinetics, a small library of known degrons was incorporated into degron-based substrates to comparatively assess their ubiquitination kinetics. The mechanism of placement of multiple ubiquitins on the different degron-based substrates was assessed by comparing experimental data to computational models incorporating first order reaction kinetics. Both multi-monoubiquitination and polyubiquitination models were considered to better understand the measured ubiquitination kinetics. Additionally, the multi-monoubiquitination model was able to capture the rate of degron-based substrate ubiquitination relative to peptide degradation by intracellular peptidases. Further, the importance of the location and proximity of the ubiquitination site lysine was determined in the three best performing degron-based substrates. Finally, experiments were conducted to assess the dependence of substrate ubiquitination on E3 ubiquitin ligase activity and preliminary work was performed to evaluate the specificity of the degron-based substrates with respect to their target E3 ligases. Ultimately, this work identified three candidate portable degrons that exhibit a higher rate of ubiquitination compared to peptidase-dependent degradation, a desired trait for a proteasomal targeting motif.

## Materials and Methods

### Substrate Synthesis and Purification

Peptides were synthesized either manually or by automated solid phase peptide synthesis on a Creosalus TetrasUI peptide synthesizer using Fmoc-protected amino acids on a CLEAR-amide resin purchased from Peptide Internationals. All natural Fmoc-[N]-protected amino acids were acquired from Advanced Chem Tech. Fmoc-NH-(PEG)-COOH, Fmoc-Ser[PO(OBzl)-OH]-OH, Fmoc-Thr[PO(OBzl)-OH]-OH,and Fmoc-Lys(ivDde)-OH were purchased from EMD Biosciences. 5,6-carboxyfluorescein was obtained from Sigma. Activation of amino acids was performed with HBTU (4 eq) and HOBt (4eq) in the presence of DIPEA (5 eq) in DMF and NMP. Peptide deprotection was carried out in 2% DBU (1,8-diazobicyclie[5.4.0]undec-7-ene) and 2% piperidine in DMF for 2 cycles of 15 min each. In cases where the peptide contained aspartates, deprotection was carried out in a 20% piperidine and 0.1 M HOBt solution in DMF for 2 cycles of 15 min each. Each natural amino acid coupling step was performed twice for 30 min or 1 h. For coupling of Fmoc-NH-(PEG)-COOH (4 eq), Fmoc-Lys(ivDde)-OH (4 eq), Fmoc-Ser[PO(OBzl)-OH]-OH (4 eq), and Fmoc-Thr[PO(OBzl)-OH]-OH (4 eq) standard coupling agents were used for a single coupling of 4 h. All peptides were acetylated at the N-terminus with 5% acetic anhydride and 6% 2,6-lutidine in DMF for 35 min. Deprotection of Fmoc-Lys(ivDde)-OH side chain was performed with 3% anhydrous hydrazine in DMF for 5×3 min. Removal of the ivDde was confirmed by the Kaiser test. Conjugation of 5,6-carboxyfluorescein (4 eq) was done using PyBOP (4 eq), HOBt (4 eq), and DIPEA (4 eq) in DMF and allowed to react overnight in the dark. Cleavage of the peptide from the resin was performed in 95∶2.5∶2.5 TFA: TIPS: H_2_O for 3 h. TFA was evaporated and cleavage products were precipitated with cold ether. The peptide was extracted into water and lyophilized. It was then purified by reverse-phase HPLC using an Atlantis C-18 semi-preparative column first with a gradient of 0 to 100% B over 60 minutes and second with a gradient of 0 to 100% B over 100 minutes, where solvent A was 95∶5 water: acetonitrile with 0.1% TFA and solvent B was 95∶5 acetonitrile: water with 0.1% TFA. After purification the peptide was lyophilized to a powder and identified using ESI or MALDI mass spectrometry.

### Cell Culture and HeLa S100 Cytosolic Lysate Generation

HeLa S3 cells (ATCC) were maintained in Dulbecco’s modified eagle medium (DMEM) with 10% v/v bovine calf serum (HyClone) and maintained in a 37°C, 5% CO_2_ environment. HeLa, THP-1, U937, and HL-60 cells were all obtained from the Lineberger Tissue Culture Facility at the University of North Carolina at Chapel Hill. HeLa cells were grown in DMEM with 10% v/v fetal bovine serum (FBS, Atlanta Biologics). THP-1 cells were grown in RPMI 1640 supplemented with 10% v/v FBS and 0.05 mM 2-mercaptoethanol. U937 cells were cultured in RPMI 1640 with 10% v/v FBS. HL-60 cells were grown in RPMI 1640 with 10% v/v FBS and 1% v/v penicillin-streptomycin. All media components are from Cellgro unless otherwise noted. Unless otherwise noted all reagents used in following assays are from Sigma-Aldrich. HeLa S100 cytosolic lysates were generated from cells based on the Dignam protocol as previously described [22]. Briefly, HeLa S3 were grown in suspension in a 3 L spinner flask until a final density of 7×10^5^ cells/mL in suspension growth media (RPMI 1640 supplemented with 5% v/v bovine calf serum) in a 37°C, 5% CO_2_ environment. Cells were then washed with ice cold PBS and pelleted at 1200×g for 10 min to calculate the packed cell volume (PCV). The cell pellet was then swelled in 5×PCV hypotonic buffer (10 mM HEPES pH 8.0, 10 mM KCl, 1.5 mM MgCl_2_, and 1 mM DTT) for 10 min at 4°C and then centrifuged at 1800×g for 10 min. Resulting cells were resuspended at 2×PCV in hypotonic buffer and then lysed with approximately 10 strokes in a Dounce glass homogenizer and centrifuged for 10 min at 1200×g. The supernatant was harvested and supplemented with 0.11×PCV of buffer B (0.3 M HEPES pH 8.0, 1.4 M KCL,and 30 mM MgCl_2_) and centrifuged at 100,000×g for 60 min at 4°C. The supernatant was then dialyzed twice, first overnight and second for 4 h, in buffer D (20 mM HEPES pH 8.0, 100 mM KCl, 0.2 mM EDTA, 0.5 mM PMSF, 1 mM DTT, and 20% v/v glycerol) at 4°C. The resulting suspension was further centrifuged at 33,000×g for 20 min at 4°C. Isolated cytosolic lysates were then quantified with a Nanodrop 2000 (Thermo Scientific), aliquoted, and stored at −80°C.

### *In vitro* Ubiquitination Assay

The *in vitro* ubiquitination assay was carried out at the indicated times at 37°C in a total reaction volume of 25 µL containing assay buffer (10 mM Tris-HCl pH 7.6 and 5 mM MgCl_2_) with 2 mM DTT, 20 µg/mL ubiquitin aldehyde (Boston Biochem), 400 µg/mL methylated ubiquitin (Enzo Life Sciences), 1×ATP energy regenerating solution (ATP-ERS, Boston Biochem), 100 µM of the proteasome inhibitor MG-132 (EMD Chemicals), 2 mg/mL HeLa S100 cytosolic lysates as the source of E1, E2, and E3 enzymes, 750 ng of indicated peptide substrate, and inhibitors Complete ULTRA and PhosSTOP (Roche). Reactions were halted by addition of 2× tricine sample buffer (Bio-Rad) and subsequent boiling. Samples were loaded onto SDS page gels (precast 16.5% Mini PROTEAN Tris-Tricine, Bio-Rad) using 1× tris-tricine running buffer and visualized with a Typhoon Imager (GE Healthcare Life Sciences). Gels were quantified using ImageJ (US National Institute of Health) by comparing sample intensity to unreacted parent peptide intensity, which is not depicted in the gels.

The cell-free ubiquitination assay was carried out in a total reaction volume of 20 µL, containing assay buffer (10 mM Tris-HCl pH 7.6 and 2 mM MgCl_2_) with 2 mM DTT, 100 µM ubiquitin 1×ATP-ERS, 1 µM E1 enzyme (Ube1, Enzo Life Sciences), 10 µM E2 enzyme (UbcH5b, Enzo Life Sciences), 1 µM Hdm2 (Enzo Life Sciences), and 10 µM peptide. Vials were incubated for 2 hours at 30°C before halting the reaction with the addition of 2× tricine sample buffer (Bio-Rad) and subsequent heating at 95°C. Samples were run on gels and imaged as described above.

### Ubiquitin Pull Down Assay

Ubiquitin pull down assays were performed similarly to the *in vitro* ubiquitination assay as described above, with the exception of a total volume of 100 µL and 4.2 µg of peptide substrate. At the end of the indicated times, samples were incubated with Control-Agarose beads (LifeSensors), diluted in TBS-T buffer (20 mM Tris-HCl pH 8.0, 150 mM NaCl, and 0.1% v/v Tween-20), for 60 min on a tube rotator at 4°C. Samples were subsequently centrifuged at 1800×g for 5 min to pellet and remove control beads. The supernatant was transferred to solution of Agarose-TUBES1 (LifeSensors) diluted in TBS-T and incubated overnight on a tube rotator at 4°C. Ubiquitin-bound beads were washed 5× with TBS-T and then the samples were eluted off of the bead with 2× tricine sample buffer, boiled for 5 min, and then isolated by centrifugation for 5 min at 13000×g. Samples were loaded onto an SDS PAGE gel, electrophoresed, visualized and quantified as described above.

### Cell Lysate-based Ubiquitin Pull Down Assay

Prior to the ubiquitination assay, cell lysates had to be harvested. Briefly, ∼1.3×10^7^ cells were centrifuged for 2 minutes at 800×g (adherent cells were first removed from the flask with Trypsin-EDTA) and then washed 2× with PBS. The cell pellet was re-suspended in MPER lysis buffer (Fisher) and vortexed for 10 minutes at room temperature followed by 4°C centrifugation for 15 minutes at 14,000×g. The supernatant was collected, quantified with a Nanodrop, and placed on ice for experimentation. The ubiquitin pull down assay was performed as described above with a total reaction volume of 100 µL except with 400 µg/mL ubiquitin (Enzo Life Sciences) instead of methylated ubiquitin. Samples were loaded onto SDS page gels (precast 4–15% TGX gels, Bio-Rad) with 1× Tris-Glycine running buffer and visualized as described above.

### Kinetic Analysis of Substrate Ubiquitination

The mathematical model for substrate ubiquitination is described in detail Text S1 in File S1. The series of ordinary differential equations were solved in MATLAB using ODE15s and the boundary conditions defined in equations S1–S6. Kinetic rate constants were determined using a Markov Chain Monte Carlo (MCMC) algorithm in MATLAB by minimizing the cumulative sum of the squared deviations (cSSD), as defined by equations S8–S9, between the calculated and experimental data similar to previous work by Cirit *et. al*. [23].

## Results

### Degron-based Substrate Design, Synthesis, and Validation

**Figure 1:**
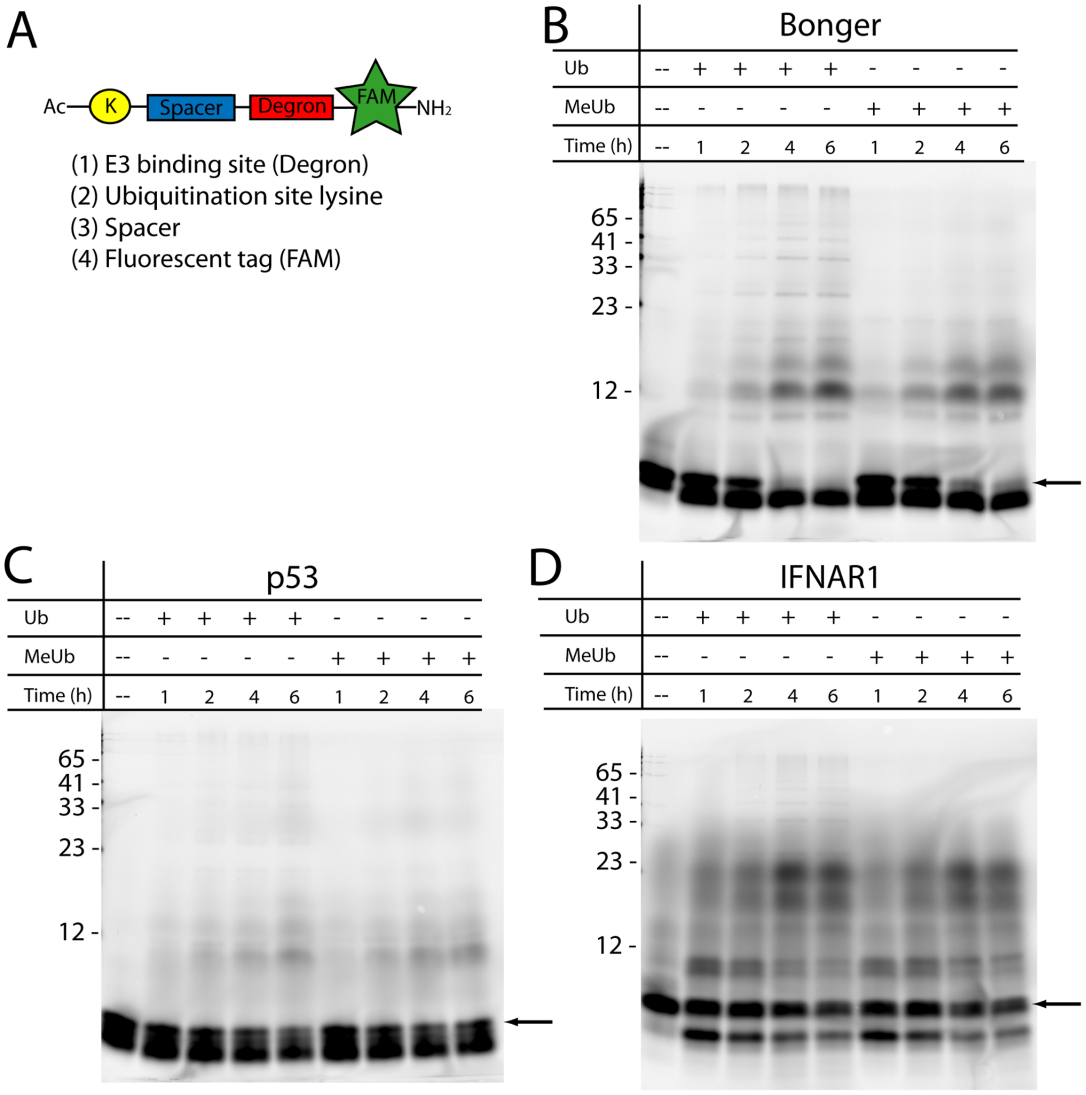
Preliminary validation of degron-based substrate ubiquitination. (A) Schematic of the degron-based substrate consisting of four essential components: the E3-ligase recognition site (degron), a proximal lysine to be ubiquitinated, a short spacing region so that the degron-bound E3 ligase can access the lysine, and a fluorescein tag for visualization. Three substrates, Bonger-based (B), p53-based (C), and IFNAR1-based (D) were incubated with either ubiquitin (Ub) or methylated ubiquitin (MeUb) in an *in vitro* ubiquitination reaction mixture for varying times. Unmodified substrate was loaded into the first lane of each gel to compare unreacted vs. degraded substrate. Arrow indicates unmodified substrate.

After the initial discovery of the ubiquitin proteasome system by Nobel laureates Ciechanover, Hershko, and Rose in 1979 [24] the number of known E3 ubiquitin ligases and their targets has expanded considerably. As such, there are a significant number of studies focused on isolating and identifying various degrons recognized by different E3 ligases from which to draw in the design of degron-based substrates. After an extensive literature search, eight degrons were selected encompassing the various classes of degrons: phospho-degrons (sequences from TAZ [25], IFNAR1 [26], SRC3 [27], and Cyclin D1 [28]), oxygen-dependent degrons (sequences from HIF-1α [29]), synthetic degrons (Bonger [8]) and degrons from essential proteins including iNOS [30] and the tumor suppressor p53 [31]. Other degrons chosen from proteins including CL1 [32], PDE4D [33], and MCL-1 [34] were evaluated; however, these substrates proved difficult to synthesize and so were not pursued. Once selected, the degron was incorporated in to the four-component degron-based substrate (Figure 1a) consisting of the degron, an N-terminal ubiquitination site lysine, a short amino acid sequence to separate the degron from the ubiquitination site lysine and prevent potential steric hindrance issues, and a C-terminal fluorescein tag for detection. The choice for an N-terminal lysine and a C-terminal fluorescent tag was based on the success of the β-Catenin degron in multiple studies [35], [36]. The sequence for each degron-based substrate, and the known E3 ubiquitin ligase that targets it, was included in Table 1 with the degron underlined (if the precise degron was known) and the phosphorylatable residues in bold, either serines or threonines, in the case of phospho-degrons. Most of the degrons found in the literature were listed as a 4–7 amino acid sequence (e.g. Bonger and IFNAR1) while others were known to be within a larger region of a protein (e.g. p53 and HIF-1α). For the sake of consistency, the length of each degron-based substrate was normalized to be ∼25 amino acids (the length of the p53 degron). To accomplish this for the smaller degrons identified, the complete sequence for each protein was obtained using the NCBI database and the N- and C-terminal regions surrounding the degron were incorporated into the degron-based substrate to achieve the desired peptide length. If the sequence did not show a proximal lysine residue to the degron (as is the case for the SRC3 and iNOS degrons) a polyethylene glycol (PEG) monomer was inserted between the sequence and an N-terminal lysine residue. A triple lysine C-terminus was added to each sequence to enhance solubility by providing a net positive charge and minimize steric issues between the bulky fluorescein tag and the amide resin used for peptide synthesis. The degron-based substrates were all synthesized using solid phase peptide synthesis (SPPS), purified by reverse phase HPLC, and verified using MALDI-TOF. Finally, a Nanodrop 2000 (Thermo Scientific) was used to measure the fluorescence intensity of all the peptides to determine both the peptide concentration and ensure that each peptide exhibited the same level of basal fluorescence. The final product was a small peptide substrate, containing the portable degron, which could be ubiquitinated.

**Table 1:** Library of degron-based substrates.

Parent Protein	E3 Ligase	Peptide Sequence	Reference
Bonger	Unknown	KTRGVEEVAEGVVLLRRRGNK(FAM)KKK	8
TAZ	SCF^βTrCP^	KPFLNGGPYHSREQ**S**TD**S**GLGLGSYK(FAM)KKK	21
HIF-1α	VHL	ASADLDLEALAPYIPADDDFQLRK(FAM)KKK	25
iNOS	Unknown	K-(PEG)-KEEKDINNNVKKTK(FAM)KKK	26
SRC3	SPOP	K-(PEG)-DVQKADV**SS**TGQGIDSK(FAM)KKK	23
Cyclin D1	SCF^FBX4^	KAAEEEEVSLAS**T**PTDVRDVDIK(FAM)KKK	24
IFNAR1	SCF^βTrCP^	KKYSSQTSQD**S**GNYSNK(FAM)KKK	22
p53	Mdm2	KPLSSSVPSQKTYQGSYGFRLGK(FAM)KKK	27
β-Catenin	SCF^βTrCP^	KAWQQQSYLD**S**GIH**S**GATTTAPK(FAM)KKK	31

Substrates were named based on the protein they were derived from, with the exception of Bonger which is a synthetic degron. Underlined regions indicate the degron while bold amino acids represented phosphorylated residues in phospho-degrons. The N-terminus of all peptides were acetylated and the C-terminus of all peptides were amidated.

Next it was essential to confirm that the degron-based substrates could be ubiquitinated. Since the library of degrons are recognized by a variety of E3 ubiquitin ligases, an assay was devised that utilized HeLa S100 cytosolic lysates containing an array of E1, E2, and E3 enzymes that recognize and ubiquitinate each of the substrates. This system not only allowed for an accurate comparison of degron performance in a single cell line, but also was compatible with degrons (e.g Bonger and iNOS) with unknown E3 ligases that could not be studied in a typical ubiquitination assay with purified enzymes. Additionally, either exogenous ubiquitin (Ub) or methylated ubiquitin (MeUb) was incorporated into the assay. Native ubiquitin enabled the formation of polyubiquitin chains while MeUb eliminated polyubiquitin chain formation by blocking each lysine residue with a methyl group. The incorporation of the fluorescein tag into the degron-based substrate enabled facile and sensitive detection. Each of the degron-based substrates was evaluated using this ubiquitination assay across multiple time points and imaged using gel electrophoresis to identify substrate ubiquitination (Figure 1). All of the substrates demonstrated increased ubiquitination with increased reaction time. Some substrates such as the Bonger-based substrate showed prominent mono-ubiquitination (Figure 1b, band at ∼12 kDa) while other substrates such as the p53-based substrate (Figure 1c) demonstrated a lesser degree of mono-ubiquitination; however these results do indicate that both substrates can be mono- and polyubiquitinated. Surprisingly, multiple bands at higher molecular weights were observed using both Ub and MeUb, as shown with IFNAR1-based substrate (Figure 1d). The higher molecular weight bands could result from either multi-monoubiquitination at several lysine residues or polyubiquitination utilizing endogenous ubiquitin found in the S100 lysates. Additionally, it is important to note the pronounced band at a molecular weight lower than that of the unmodified substrates (Figure 1b–d) was most likely due to substrate degradation by cellular peptidases [37]. The remaining five degron-based substrates yielded similar results to that of the Bonger, p53 and IFNAR1-based substrate, although with varying degrees of ubiquitination and band intensity (data not shown). These results suggested that the degron-based substrates were, in fact, ubiquitinated.

### Quantification of Degron-based Substrate Performance Demonstrates Variable Degrees of Ubiquitination

**Figure 2:**
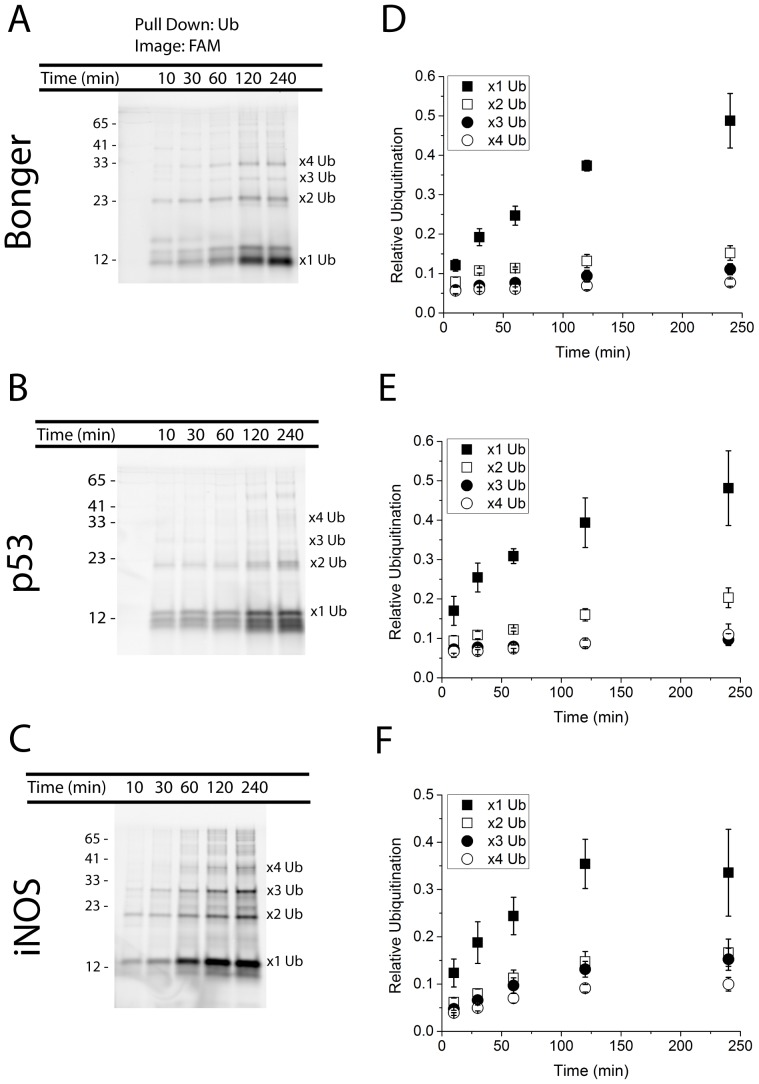
Quantification of substrate ubiquitination. Eight members of the substrate library were subjected to a ubiquitin pull down assay to confirm substrate ubiquitination. Ubiquitin-conjugated substrates were purified using ubiquitin-binding beads and then visualized using the fluorescein tag. Three substrates are depicted: Bonger-based (A), p53-based (B), and iNOS-based (C) substrates. Relative protein sizes are compared to values obtained from a fluorescent protein marker (numerical text on the left of A–C). Suspected mono-, di-, tri-,and tetra-ubiquitin conjugated substrates are labeled accordingly. The fluorescence intensity of the bands was measured for Bonger (D), p53 (E), and iNOS (F) -based substrates. The data points represent the average (n = 3) and the error bars the standard deviation of the data points.

To verify that the previous observations corresponded to ubiquitinated substrate, a ubiquitin pull-down assay was performed. Substrates were reacted with the ubiquitination reaction mixture above and then incubated with ubiquitin agarose beads [38]. As a result, ubiquitin-bound substrates were separated from non-ubiquitin-bound substrates and degraded substrates. For these assays only methylated ubiquitin was used to permit a more precise quantification of substrate ubiquitination. While it is well known that polyubiquitination is the hallmark modification targeting a protein or peptide to the 26S proteasome for degradation, incorporation of wild type ubiquitin into the ubiquitin pull down assay results in an abundance of observable bands on the gel which would require a more complex analysis of substrate ubiquitination. To simplify the analysis, methylated ubiquitin was used instead of wild type ubiquitin. While this change could impact the ability of the substrate to be targeted to the proteasome, this was not an issue for these experiments since the proteasome was already inhibited by the inhibitor MG-132. The samples were then separated by gel electrophoresis and imaged using the fluorescein tag so that only fluorescent, ubiquitinated peptides were detected. Control experiments were performed to confirm that the findings were not the result of non-specific binding between the degron-based substrate and agarose beads or due to fluorescence of native proteins in the S100 lysates (data not shown). All eight degron-based substrates exhibited time-dependent ubiquitination (Figure 2 & Figure S1 in File S1). The iNOS-based (Figure 2c), Bonger-based (Figure 2a), and p53-based (Figure 2b) substrates showed the most intense bands at 240 min. A marked decrease in fluorescent intensity was observed at the 240 min time point for the remaining five substrates, with the SRC3 and Cyclin D1 degrons exhibiting the weakest fluorescent signal and, hence, the lowest degree of ubiquitination. In order to contrast performance between the substrates, the fluorescent intensity of each experimental time point was compared to the intensity of the unmodified substrate which was not exposed to the ubiquitination assay. The p53-based substrate exhibited the greatest ubiquitination (Figure 2e), while Bonger and iNOS-based substrates both demonstrated higher ubiquitination (Figure 2d and 2f respectively) when compared to the remaining five degrons (Figure S1 in File S1). Taken together, these results indicate while all of the degron-based substrates were ubiquitinated, the degree of ubiquitination was highly variable and depended on the degron.

Further inspection of the gels showed the presence of additional bands at ∼21, ∼30, and ∼39 kDa, each corresponding to di-, tri-, and tetra-ubiquitinated substrates (Figure 2). While some degrons exhibited an equal preference for mono- *vs.* di-ubiquitinated states (e.g. the HIF-1α degron-based substrate, Figure S1c) others display a preference for di- and tri-ubiquitinated states such as the TAZ (Figure S1a in File S1) and Cyclin D1-based substrates (Figure S1d in File S1). Since methylated ubiquitin was calculated to be in substantial excess (>100X) relative to that of native ubiquitin, the formation of polyubiquitin chains was unlikely [39]. To ensure that the higher molecular weight species correspond to multiple ubiquitins present on a single peptide, two degron-based substrates were exposed to exogenous ubiquitin, instead of MeUb. These results (Figure S2 in File S1) demonstrated that the mono-, di-, tri-, and tetra-ubiquitinated species migrate similarly on the gel in the presence of either MeUb or exogenous Ub. Based on this, it was likely that the presence of higher molecular weight species, in the presence of MeUb, were the result of the addition of multiple MeUbs on the degron-based substrates due to the presence of greater than one lysine on the substrates.

### Kinetic Analysis Suggests Degron-based Substrate Multi-monoubiquitination

Once it was determined that the degron-based substrates could be ubiquitinated, the next step was to calculate the ubiquitination kinetics of each of the degrons to establish which degrons were ideal candidates to incorporate into a proteasome reporter. To accomplish this objective, a kinetic model was developed to describe the step-wise ubiquitination of the degron-based substrates. The goal of this model was to both determine the reaction kinetics for ubiquitin transfer to the degron-based substrates and distinguish polyubiquitination from multi-monoubiquitination. This model incorporated first order reaction kinetics to describe the transfer of each ubiquitin to the substrate and included both unmodified and ubiquitin-bound substrate degradation by peptidases based on the results observed in Figure 1b–d. All degradation terms incorporated into the model are due to endogenous proteases and peptidases found in the S100 lysates since the 26S proteasome was inhibited by the addition of the proteasome inhibitor MG-132. Each step in the reaction was described by a corresponding rate constant (k_1_–k_9_). Due to the presence of the deubiquitinating enzyme (DUB) inhibitor ubiquitin aldehyde, substrate deubiquitination was neglected. Further, the proteasome inhibitor MG-132 was added at an appropriate amount to neglect proteasome-mediated degradation of the substrates. To accurately fit the model to the quantitative data, a Markov Chain Monte Carlo algorithm was utilized to successively minimize the cumulative sum of the squared deviation between the model and the data obtained from the ubiquitin pull down assays incorporating methylated ubiquitin (Figure 2a–c) at each time point for all four ubiquitinated species (Equation S8–S9 in File S1) [23]. It was decided to use the MeUb pull down data to fit the model to ensure the bands observed did in fact correspond to ubiquitinated substrate. The governing equations, boundary conditions and an explanation of model development are described in more detail in Text S1 in File S1. The initial model developed was based on substrate polyubiquitin chain formation (Figure S3a in File S1) and failed to recapitulate the data observed in Figure 2a from the Bonger-based substrate. This model (Figure S3b in File S1) strongly suggested that the higher molecular weight bands observed in Figure 2a were not the result of polyubiquitinated substrates.

**Figure 3:**
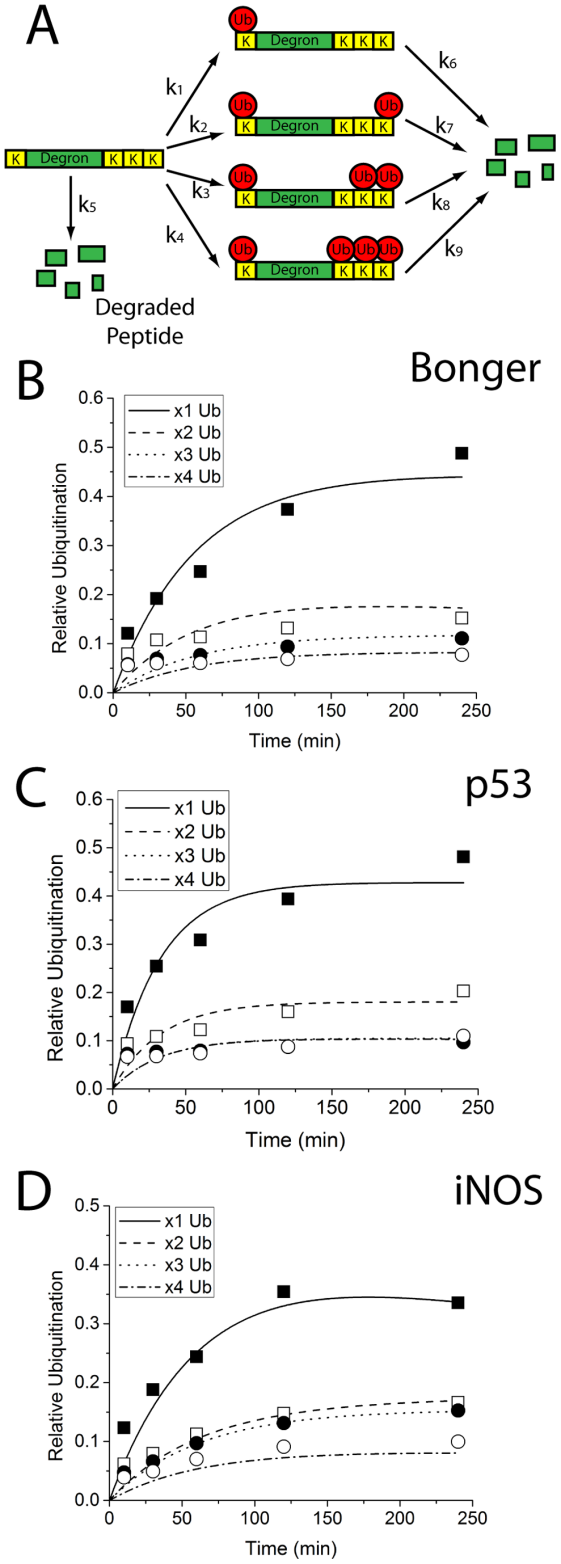
Kinetic analysis of substrate ubiquitination implicates multi-monoubiquitination of degron-based substrates. (A) Schematic representing proposed model for multi-monoubiquitination of the degron-based substrates. Rate constants correspond to Equations S1–S6 representing substrate ubiquitination and peptidase-dependent degradation. Model output (lines) for the three degron-based substrates: Bonger (B), p53 (C), and iNOS (D) demonstrates a good fit between model prediction and observed data. Model fit for mono-, di-, tri-, and tetra-ubiquitinated substrates are labeled accordingly. Model output is depicted as relative ubiquitination (C_i_/C_o_) to permit comparison across substrates (Eq. S7). Multiple ubiquitins conjugated to a substrate are denoted by according to the legend in the upper left corner.

A second model based on substrate multi-monoubiquitination was developed and evaluated. This model assumed that the generation of di-, tri-, and tetra-ubiquitinated species was the result of multiple methylated ubiquitins being conjugated to the multiple available lysines found on the degron-based substrate (Figure 3a). As opposed to the polyubiquitination model, the multi-monoubiquitination model accurately fit the data for all eight degron-based substrates (Figure 3b–d, Figure S4 in File S1, Table 2). Using this model, kinetic rate constants for all ubiquitination (k_1_ for mono-, k_2_ for di-, k_3_ for tri-, and k_4_ for tetra-) and peptidase-dependent degradation steps (Table 2) were calculated. The multi-monoubiquitination model nicely corresponded to the observed results in Figure 2. The p53 and Bonger-based substrates exhibited the most rapid mono-ubiquitination kinetics, while the SRC3 and HIF-1α-based substrates exhibited the slowest kinetics, with the remaining five falling in between. The model also captured the preference for di-ubiquitination of the Cyclin D1 degron (k_2_ is two-fold greater than k_1_) and the equal preference of mono- and di-ubiquitination states found in the HIF-1α degron (k_1_ of 2.6 and k_2_ of 3.0). Further, the similar behavior and observed kinetics for IFNAR and TAZ degrons were mostly likely due to the fact that these degrons are recognized by the same E3 ligase, SCF^βTrCP^ (Table 1). To test the robustness of the multi-monoubiquitination model, a ninth substrate based on the degron from the protein β-Catenin was assessed. This substrate was selected because it is ubiquitinated by the same E3 ligase as TAZ and IFNAR1, SCF^βTrCP^. The model accurately fit the data for the multi-monoubiquitination of the β-Catenin degron-based substrate with similar rate constants compared to the TAZ and IFNAR1-based substrates (Table 2). Finally, it was important to assess the validity of the model by addressing the relative goodness of fit between the experimental data and the model predictions as determined from the multi-monoubiquitination model. For this particular analysis it was decided to calculate the sample Pearson correlation coefficient (Eq. S10 in File S1), a widely used metric to measure the dependence between two variables. A correlation coefficient was calculated between the model predictions and experimental data for mono-, di-, tri-, and tetra-ubiquitinated substrate for all nine degron-based substrates (Table S1 in File S1). As per convention, a positive value approaching 1 corresponds to a very good fit, which is observed for all nine substrates indicating a good fit between model and data. Additionally, the worst fit was observed for the Cyclin D1-based substrate, which tracks nicely with the cSSD value obtained from the MCMC algorithm.

**Table 2:** First order rate constants determined by kinetic analysis of degron-based substrates.

(1/min)*10^3^	Bonger	p53	iNOS	IFNAR1	TAZ	Cyclin D1	SRC3	HIF-1α	β-Catenin
k_1_	8.1	13.1	6.8	7.2	6.1	3.4	2.8	2.6	6.2
k_2_	3.6	5.5	2.7	5.2	7.2	5.9	1.5	3	6.4
k_3_	2.1	3.2	2.5	2.1	4.7	4.1	1.2	1.9	2.6
k_4_	1.5	3.2	1.4	1.2	2.2	2.1	1.0	1.0	1.4
k_5_	4.0	8.7	4.2	7.5	2.3	7.2	8.5	7.0	4.0
k_6_	0.01	0.01	1.0	0.01	1.5	0.01	0.01	0.1	0.1
k_7_	1.0	0.01	0.01	0.01	0.1	0.01	0.01	0.1	0.1
k_8_	0.01	0.01	0.01	0.01	0.5	0.01	0.01	0.4	1.0
k_9_	0.01	0.33	0.3	0.1	0.1	1.8	0.33	0.8	3.0
k_1_/k_5_	2.03	1.51	1.62	0.96	2.65	0.47	0.33	0.37	1.55
cSSD	2.988	3.444	2.431	2.802	4.495	4.184	1.897	1.689	2.203

Rate constants for ubiquitination (k_1_–k_4_) and peptidase-dependent degradation (k_5_–k_9_) determined by Markov Chain Monte Carlo algorithm solving the series of kinetic differential equations modeling substrate ubiquitination (Equations S1–S6). Sum of squared difference calculated for each substrate according to Equation S8–S9. Values contained in table are multiplied by 10^3^.

While the experimental data did not provide information regarding the peptidase-dependent degradation of ubiquitinated and unmodified substrates (due to the limitations of the pull down assay), the model was able to provide insight into which species were likely to be the most rapidly degraded. Across all degron-based substrates, the most dominant degradation term was k_5_, corresponding to the degradation of unmodified parent (Table 2). This term, sometimes two orders of magnitude greater than other degradation terms, indicated that degradation of unmodified substrate was the preferred mode of peptide degradation under these experimental conditions. Finally, since the goal of this analysis was to isolate portable degrons capable of rapid ubiquitination, it was important to calculate a metric that compared not only substrate ubiquitination, but also accounted for the substrate resistance to intracellular peptidases. As such, a comparison of mono-ubiquitination vs. degradation was determined by relating the two rate constants governing these reactions (k_1_/k_5_). Three substrates based on the degrons from Bonger, p53, and iNOS all exhibited a ratio in favor of ubiquitination (k_1_/k_5_>1), while other substrates based on the degrons from SRC3 and HIF-1α demonstrated a shift in favor of degradation (k_1_/k_5_<1) as summarized in Table 2. Additionally, while the k_1_/k_5_ value for the TAZ-based substrate argued for favorable ubiquitination, it was neglected from further study due to the relatively poor model fit (Table 2) and high degree of variability in substrate ubiquitination (Figure S1a in File S1).

### Different Degrons Exhibit a Locational Preference for the Ubiquitination Site Lysine

As the multi-monoubiquitination model provided valuable insight in the kinetics of the degron-based substrate, the next step was to further characterize the top three performing degrons by evaluating the optimal length of the degron-based substrate and identify the preferred location of the ubiquitination site lysine. When incorporating a degron into a proteasome reporter, a single ubiquitination site lysine is desirable to ensure substrate polyubiquitination (and not multi-monoubiquitination) since this modification is what targets the reporter to the proteasome. Additionally, it was important to determine if the substrates absolutely required the spacing amino acids between the degron and the ubiquitination site lysine or if the portable degron alone was enough to illicit ubiquitination. To accomplish these objectives a series of modified substrates were synthesized based on the Bonger, p53, and iNOS degrons, since these degron-containing substrates exhibited the highest k_1_/k_5_ value (Table 2). Each of these substrates was assayed using the ubiquitin pull down method supplied with MeUb to show a single ubiquitinated species.

**Figure 4:**
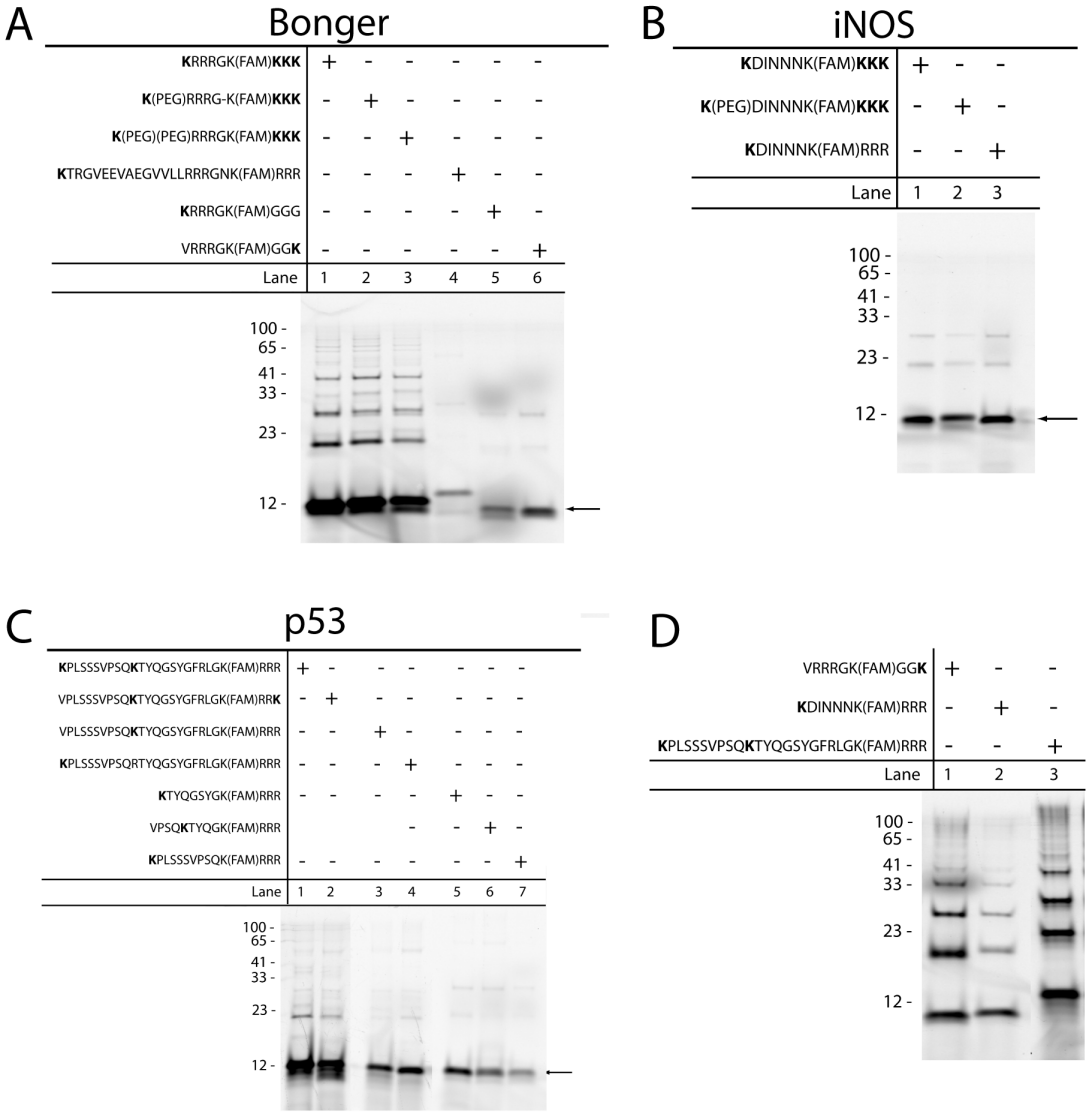
Effect of ubiquitination site lysine location relative to the degron. Modified versions of the substrates based on the Bonger (A), iNOS (B), and p53 (C) degrons were incubated in the *in vitro* ubiquitination pull down assay mixture with MeUb, purified and electrophoresed. (D) The same assay was conducted using native ubiquitin and the modified substrates based on Bonger (lane 1), iNOS (lane 2) and p53 (lane 3) degrons. All reactions were incubated for 2 hours at 37°C prior to continuation with the pull down assay.

Since the degron was already identified for the Bonger substrate (RRRG), this substrate was initially assessed. First, the importance of proximity between the degron and the ubiquitination site lysine was evaluated by designing substrates without the 15 amino acid linking sequence between the degron and the N-terminal lysine (Figure 4a, lane 1) or with the linking sequence replaced by one or two PEG monomers (Figure 4a, lane 2–3 respectively). These initial substrates still contained the triple lysine C-terminus to determine if the spacing could abrogate substrate multi-monoubiquitination. Deletion of the spacing sequence did not negatively impact the ability of the substrate to become ubiquitinated, indicating that the Bonger degron only required the four amino acids to be ubiquitinated. Next, the ideal position of the lysine residue in both full length (Figure 4a, lane 4) and shortened (Figure 4a, lanes 5–6) Bonger-derived substrates were assessed by replacing the C-terminal triple lysine chain with either a triple arginine or triple glycine sequence. The selection of amino acid substitution was based on a net overall positive charge to ensure proper peptide synthesis. Both of these substrates exhibited a marked decrease in ubiquitination, which suggested that the preferential lysine location was not on the N-terminus, but on the C-terminus. These results correspond to what was previously observed by the researchers who identified the Bonger degron, that the C-terminal placement of the degron was essential to degradation [8]. A final peptide was synthesized containing only the degron with an N-terminal V→K substitution and a C-terminal lysine (Figure 4a, lane 6). Valine was selected due to its relatively high half-life based on the N-end rule [40]. This substrate demonstrated significantly greater ubiquitination relative to those containing an N-terminal lysine suggesting that this degron performed best with a C-terminal lysine. The reduced ubiquitination of the Bonger-based substrate with a single lysine residue compared to Bonger-based substrates with multiple lysines (compare Figure 4a lane 1 to lane 6) was most likely due to multiple mono-ubiquitinated species (e.g. MeUb conjugated to either the N-terminus or the C-terminus). A series of similar sequence modifications were also performed using the known iNOS degron (DINNN). For this degron, removal of the spacing sequence between the degron and the N-terminal lysine residue eliminated the presence of multi-monoubiquitinated species (Figure 4b, lane 1), a phenotype which could not be rescued by the substitution of the spacing sequence with a PEG monomer (Figure 4b, lane 2). Replacing the triple lysine C-terminus with a triple arginine sequence revealed that the N-terminal lysine residue was the preferred ubiquitination site for this degron (Figure 4b, lane 3).

The analysis of the p53-derived substrate was more complicated since the shortest portable degron sequence was not known. As such, instead of starting with the smallest known degron, as with the Bonger and iNOS sequences, this analysis began by investigating the preferential location of the lysine residue using a similar replacement strategy as described above. Both N- and C-terminal ubiquitination site lysines were strongly mono-ubiquitinated, with a small degree of di-ubiquitination (Figure 4c, lanes 1–2). The presence of the di-ubiquitinated substrate was most likely due to ubiquitination of the internal lysine residue, which was initially not removed in case it was an essential part of the degron. Next, the role of the internal lysine residue as the preferential ubiquitination site lysine was considered by comparing an N-terminal lysine (Figure 4c, lane 3) with the internal lysine residue (Figure 4c, lane 4). Both of these iterations yielded similar levels of ubiquitination but no di-ubiquitination was observed, suggesting that either lysine was sufficient for mono-ubiquitination. Finally, preliminary studies into the isolation of the smallest portable degron based on p53 were performed. Randomly selected sequences from the full length degron-based substrate were incorporated into three peptides including the middle (Figure 4c, lane 5–6) and N-terminal (Figure 4c, lane 7) regions of the p53 degron. The shortened sequence incorporating the internal lysine residue as the ubiquitination site lysine exhibited the strongest degree of ubiquitination (Figure 4c, lane 5–6). These results indicate that only a portion of the previously established p53 degron was absolutely required for ubiquitination.

Lastly, polyubiquitination of the optimized substrates, using native ubiquitin in the ubiquitin pull down assay, was carried out to determine whether the altered sequences negatively impact substrate polyubiquitination. The C-terminal lysine-containing Bonger-based substrate (Figure 4d, lane 1), the N-terminal lysine-containing iNOS-based substrate (Figure 4d, lane 2), and the N-terminal lysine-containing p53-based substrate (Figure 4d, lane 3) were evaluated. All three substrates showed successful polyubiquitination, although to varying degrees most likely dependent on the differing activities of the E3 ubiquitin ligases in the HeLa S100 substrates. Additionally, polyubiquitination of the optimized substrates derived from the Bonger, iNOS, and p53 degrons was compared to polyubiquitination of the initial full length substrates described in Table 1. All were polyubiquitinated in a similar manner indicating that shortening the substrate or changing the lysine location did not negatively affect the recognition or performance of the degron (data not shown).

### Selected Degron-based Substrates Exhibit Variable Ubiquitination Rates in Multiple Cell Lines due to Differences in E3 Ligase Activity

**Figure 5:**
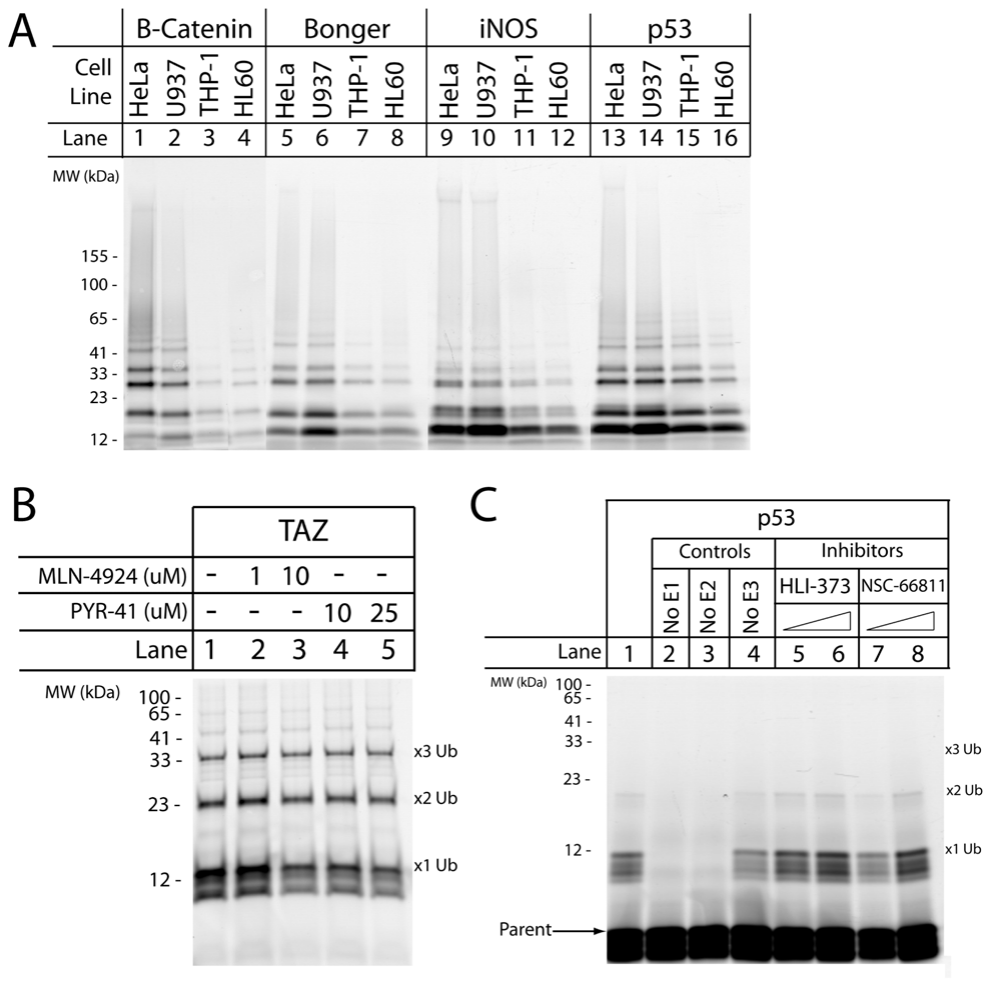
Variations in E3 ligase activity result in fluxuations in degron-based substrate ubiquitination. (A) To evaluate the impact of different levels of E3 ligase activity, the four top performing degron-based substrates (β-Catenin, Bonger, iNOS, and p53) were incubated in lysates from four different cell lines (HeLa, U937, THP-1, and HL60) in the ubiquitin pull down assay. Further, the assay was supplemented with wild type ubiquitin to examine polyubiquitin chain formation. All reaction mixtures were incubated for 2 hours at 37°C prior to continuation with the pull down assay. (B) The effects of a NEDD8 inhibitor (MLN-4924) and an E1 inhibitor (PYR-41) were examined on the TAZ-based substrate (a target of the SCF^βTrCP^ E3 ligase). The ubiquitin pull down was performed as previously described with the reactions incubated for 2 hours with the listed inhibitor concentrations at 37°C prior to continuation with the pull down assay. (C) A cell-free ubiquitination assay was performed on the p53-based substrate using an enzymatic cascade culminating with 1 µM Hdm2 and supplemented with wild type ubiquitin. Lane 1 contains all assay components, lane 2–4 were control experiments removing a member of the E1–E3 enzymatic cascade. The assay was then supplemented with two commerical Mdm2 inhibitors: HLI-373 (1,10 µM, lane 5–6 respectively) and NSC-66811 (1,10 µM, lane 7–8 respectively). All reactions were incubated for 2 hours at 30°C prior to halting the reaction with sample buffer. Arrow indicates unmodified substrate.

One complexity in designing a ubiquitin-dependent proteasome targeting sequence is the reliance on members of the enzymatic cascade responsible for substrate ubiquitination. Since the degron-based substrates described in this analysis were all derived from known degrons of known proteins targeted by E3 ubiquitin ligases (except Bonger), it was worthwhile to examine the effect of E3 ligase activity on substrate ubiquitination. Further, due to the inherent tendency for peptide-based substrates to exhibit a lack of specificity [41]–[43], it was valuable to determine if some of the degron-based substrates are indeed targeted by their cognate E3 ubiquitin ligases. Two separate routes were taken to accomplish these objectives. First, the S100 lysate-based ubiquitin pull down assay was adjusted by substituting lysates from four different cell lines for the HeLa S100 lysates. The top four performing degron-based substrates based on results from the multi-mono-ubiquitination model (β-Catenin, Bonger, iNOS, and p53) were then incubated in the adapted ubiquitin pull down assay using lysates from HeLa, U937, THP-1, and HL-60 cells. Further, to ensure that the degron-based substrates could be polyubiquitinated in these lysates, wild type ubiquitin was added to the reaction mixture (instead of the previously used methylated ubiquitin). The results of this experiment demonstrated that degron-based substrate ubiquitination is strongly related to the relative activities of the E1–E3 enzymatic cascade found in each cell line (Figure 5a). The lysates from HeLa and U937 cells exhibited similar results to what was previously observed in the S100 lysates; however substrate ubiquitination in THP-1 and HL60 cells was substantially diminished. While precise concentrations and activities of the E3 ubiquitin ligases are unknown in each of these cell lines, it can be inferred from these results that there are differences in activities which result in variations in substrate ubiquitination.

To perform a more precise analysis of the impact of variations in E3 ligase activity on degron-based substrate ubiquitination and to identify any issues with peptide specificity, the second route of experimentation was the application of known inhibitors of the E1–E3 enzymatic cascade. While there are a number of known inhibitors for the E3 ligase Mdm2, there is a pronounced lack of commercially available inhibitors for other E3 ubiquitin ligases. However, recent work in the Deshaies lab has found that protein ubiquitination by the E3 ligase SCF^βTrCP^ is strongly enhanced by the ubiquitin-like protein NEDD8 [44] and one of the commercially available inhibitors is a NEDD8 inhibitor, MLN-4924. To examine the effects of partially inhibiting SCF^βTrCP^, TAZ-based substrate ubiquitination was assessed in the presence and absence of this inhibitor (Figure 5b). It was found that ubiquitination of the TAZ-based substrate was slightly impaired in the presence of the NEDD8 inhibitor (Figure 5b, compare lane 1 vs. lane 3). Additionally, application of a general E1 enzyme inhibitor (PYR-41) resulted in reduced ubiquitination of the TAZ-based substrate. While these results do not ultimately verify the specificity of the TAZ-based substrate, they do lend credence to its targeting by the E3 ligase SCF^βTrCP^.

To assess the specificity of Hdm2-dependent ubiquitination (the human homolog of Mdm2) of the p53-based substrate, a cell-free ubiquitination assay was implemented. Results from this experiment showed that the p53-based substrate was ubiquitinated in an Hdm2-dependent manner (Figure 5c, lane 1) and that removal of the E1 or E2 enzyme halted this reaction. Interestingly, removal of Hdm2 from the reaction mixture still resulted in p53-based substrate ubiquitination, although to a lesser extent. This observation could be explained by a direct interaction between the p53-based substrate and the E2 enzyme, as removal of the E2 enzyme prevents substrate ubiquitination. Finally, two different commercially available inhibitors of Hdm2 were added to the cell-free reaction mixture to evaluate their impact on Hdm2-dependent ubiquitination (Figure 5c, lanes 5–8). No decrease in p53-based substrate ubiquitination was observed in response to HLI-373, however a 1 µM dose NSC-66811 resulted in a pronounced decrease in substrate ubiquitination (Figure 5c, lane 7).

## Discussion

Novel substrates capable of intracellular ubiquitination are a highly successful class of reporters capable of measuring cellular proteasome activity. With the growing trend of therapeutics targeted against the proteasome, it is imperative to develop the next generation of proteasome reporters that are both rapidly targeted to the proteasome and compatible with single cells. While there exist a few successful proteasome reporters, many of these established methods are not compatible with single cells and require large populations of cells and complex genetic manipulation [21]. In recent years it has become apparent that analysis of single cells, especially those obtained directly from a patient, provide a great deal more information than analysis of bulk cell lysates due to the heterogeneous nature of tumor biopsies [45]. To address the need for single cell-compatible proteasome reporters, this study focused on the characterization and analysis of multiple degrons to identify those with rapid ubiquitination kinetics for the eventual incorporation into next generation proteasome reporters. Ultimately, three candidates were selected that show a preference for ubiquitination over peptidase-dependent degradation and further study identified the preferred location of the ubiquitination site lysine.

The library of degron-based substrates was generated using solid phase peptide synthesis, a technique which offers several advantages when compared to other methods commonly employed to create proteasome reporters including the transient and stable transfection of cells using expression vectors. The substrates were synthesized and purified on a much shorter time scale, permitting a more rapid turnaround of design iterations (as demonstrated by the results presented in Figure 4). Further, generating substrates in this manner eliminated the need for complex genetic engineering techniques which are not compatible with patient samples or small cell populations. Ultimately, reporters containing these portable degrons could be directly incorporated into patient samples either through native endocytosis or by the addition of a cell permeable sequences such as a myristol tag or the TAT sequence [46]. Measurement of substrate conversion to product (ubiquitinated substrate) could be accomplished by incorporating an environment-sensitive fluorophore on the substrate or by using a microelectrophoretic method such as capillary electrophoresis (CE) to separate and quantify the substrate and product [18]–[20].

The initial experiments performed to assess substrate ubiquitination involved a ubiquitination assay incorporating HeLa S100 lysates as the source of E1, E2, and E3 enzymes (Figure 1) supplemented with an excess of either methylated or wild type ubiquitin. While these preliminary experiments strongly implied substrate ubiquitination, it was decided to validate this result by selectively isolating ubiquitinated peptide through a ubiquitin pull down assay using agarose beads conjugated to a ubiquitin specific antibody (TUBE1). The results from the pull down assay verified that all eight substrates were being ubiquitinated (Figure 2, Figure S1 in File S1). Further, for the sake of simplifying the analysis of peptide ubiquitination, only methylated ubiquitin was used in the pull down assay instead of wild type ubiquitin. This resulted in a lower (<4) number of quantifiable bands that could ultimately be incorporated in the model. One limitation of this method is that the TUBE1 product demonstrates a pronounced preference for binding polyubiquitinated substrates when compared to mono-ubiquitinated substrates; however after working closely with the quality control department of LifeSensors, the TUBE1 product was verified to also bind mono-ubiquitinated species, although to a lower extent. Ultimately a proteasome-targeting motif must be polyubiquitinated *in vivo* to successfully target the reporter to the 26S proteasome. To ensure that the degron-based substrates described herein met this criterion, an additional ubiquitin pull down assay was performed with wild type ubiquitin to verify the substrates ability to be polyubiquitinated (Figure S2 in File S1).

The results from the ubiquitin pull down assay did verify that all eight members of the library were ubiquitinated; however, the presence of higher molecular weight bands in all of the gels suggested that the substrates were either being poly- or multi-monoubiquitinated. Due to the limitations of gel electrophoresis, it was not possible to confirm which method of ubiquitination was taking place. To address this, a kinetic model incorporating first order reaction kinetics was developed to verify substrate ubiquitination. A preliminary model based on substrate polyubiquitination failed to converge on the data (Figure S3 in File S1), suggesting that the degron-based substrates were being multi-monoubiquitinated. The abundance of methylated ubiquitin, when compared with endogenous ubiquitin found in the HeLa S100 lysates, and the presence of multiple potential ubiquitination site lysines also supported the hypothesis of multi-monoubiquitination. Further, control experiments were performed that verified that the higher molecular weight bands found in the MeUb pull down migrated at roughly the same location as the di-, tri-, and tetra-ubiquitinated species when incubated with exogenous ubiquitin (Figure S2 in File S1). The second model, incorporating multi-monoubiquitination kinetics, nicely fit the data for all eight substrates from the library, in addition to a ninth substrate derived from the β-Catenin degron, based on the relatively low cumulative sum of the squared deviation (cSSD) values obtained (Table 2). The hypothesis for multiple binding events is currently favored on account of the extended reaction times necessary for measurable ubiquitination (∼10 minutes) compared with previous results showing ubiquitin-binding on the order of seconds to minutes. The presence of methylated ubiquitin compared to ubiquitin could also impact substrate loading and favor a multiple binding event model as the kinetics of methylated ubiquitin conjugation could impact E2 loading. This inability to accurately determine substrate loading highlights a limitation associated with gel electrophoresis and emphasizes the need for more precise analytical techniques.

While there exist kinetic studies of specific E3 ligases [36], [47], [48] and individual degrons [49] there has not been a comprehensive analysis of ubiquitination kinetics of multiple degrons in a single study. The presented work allowed for the calculation of rate constants for multiple degrons in a single cell line, effectively comparing the ubiquitination kinetics of different E3 ligase targets. Additionally, a comparison of the rate constants for mono-ubiquitination vs. degraded substrate allowed for the identification of degrons that exhibit a preference for either scenario and ultimately served as the basis for the selection of which degrons to further characterize (Table 2). Further, to estimate the accuracy of the data presented here, the rate constants for the mono-ubiquitination of two substrates (based on p53 and β-Catenin) were compared to published values and it was found that the results presented in this study appear to underestimate previously reported data. Full length p53 ubiquitination was previously reported at 3 min^−1^ [47] (compared to 0.0131 min^−1^ in this study), while the rate constant for β-Catenin ubiquitination was reported as 0.28 min^−1^ [44] (compared to 0.0062 min^−1^ in this study). The discrepancy in these values can be addressed by the difference in experimental methods used to obtain the data. The p53 values were obtained only considering protein levels in the nucleus (while our values were obtained from cytosolic lysates) and the authors only looked at p53 polyubiquitination. The β-Catenin values were obtained from an optimized test tube reaction with excess amounts of E1, E2, and E3 ligases and no competing peptidases. As a result, is it not entirely unexpected for values obtained in this study from lysates to underestimated values obtained from other optimized systems.

A limitation of using ubiquitin-depending targeting sequences for the proteasome is the reliance on the E1–E3 enzymatic cascade to efficiently ubiquitinate substrate. Due to this fact, it was important to assess the impact of variations in E3 ligase activity on degron-based substrate ubiquitination. This analysis was performed by incubating the top four performing degron-based substrates in lysates from four different cells, each with its own levels of E1, E2, and E3 enzymes (Figure 5a). While all the substrates were polyubiquitinated in these lysates, the degree of ubiquitination varied greatly among cell lines. This is an important result to consider when developing a proteasome reporter using one of the degron-based substrates as the targeting sequence since the cells being targeted by this reporter will exhibit varying degrees of E3 ligase activity. While the kinetic analysis performed in the S100 lysates did identify portable degrons to incorporate in a novel reporter, additional considerations must be taken when selecting which portable degron to use for a specific cell line.

Another factor to consider when using peptide substrates as reporting tools is their tendency to exhibit a lack of specificity for their target enzymes. This attribute has been well documented using peptide substrates for target kinases [41]–[43], but has not been well addressed for E3 ubiquitin ligases. Using commercially available inhibitors or a cell-free ubiquitination assay it was demonstrated that two of the substrates based on TAZ and p53 were ubiquitinated, in part, by their target E3 ubiquitin ligases (Figure 5b–c). In the case of the TAZ-based substrate, the partial inhibition of ubiquitination by the NEDD8 inhibitor matches results obtained previously of the enhancing potential of NEDD8 for the E3 ligase SCFβTrCP [44]. The results for the Hdm2-dependent ubiquitination were more interesting. It was observed that the p53-based substrate was ubiquitinated by Hdm2; however the presence of Hdm2 was not absolutely required for substrate ubiquitination. This is a curious observation, but not an unexpected one. The goal of many E3 ligases is to bring the Ub∼E2 complex into close proximity of the target protein to allow for direct transfer of the ubiquitin from the E2 to the target protein. Recent publications have found that the E3 ubiquitin ligase only enhances substrate ubiquitination, but is not absolutely required [50]. Further, it is distinctly possible that the sequence of the p53-based substrate is similar enough to the sequence on Hdm2 (and Mdm2) to allow for ubiquitination. Ultimately, there is a general lack of concern about E3 ligase specificity towards a portable degron due to the fact that, when designing a proteasome reporter, it is not an absolute requirement that the peptide substrate express singular fidelity for one E3 ubiquitin ligase. The main concern for a proteasome reporter is that it can be rapidly and efficiently ubiquitinated. While the exact specificity of some of the degron-based substrates is not known, it does not limit their ability to be ubiquitinated and targeted to the proteasome.

One potential challenge associated with these peptide based substrates is their rapid degradation by intracellular peptidases. This phenomena was observed experimentally (Figure 1b–d) and addressed by the multi-monoubiquitination model (k_5_ in Table 2). Since all proteasome reporters evaluate enzymatic activity through the quantification of reporter degradation over time in the presence or absence of proteasome inhibitors such as MG-132 and Bortezomib, it is imperative to ensure that reporter degradation is due to the proteasome and not off target degradation by peptidases. As such, a successful proteasome reporter must be stable under normal cellular conditions to effectively measure the activity of its target enzymes. Proctor et. al. have previously established that peptidase resistance could be imparted to a kinase reporter through the substitution of native with non-native amino acids [51]. This method, though successful, requires a great deal of time to identify the cleavage sites and to optimize substrate resistance while simultaneously minimizing reporter activity. Another method, currently underway, is the incorporation of the well-folded structures to the N-terminus of peptides to impair substrate entry into the catalytic cleft of the peptidase and prevent degradation [52]–[54]. Since it was found that the degron alone was sufficient for ubiquitination of two of the degron-based substrates assessed in this study (Figure 4), it is highly feasible to incorporate this N-terminal protecting domain to increase intracellular peptidase resistance.

Finally, while the p53 substrate was previously identified as an essential region for Mdm2-mediated ubiquitination [31], the precise portable degron and the preference of the ubiquitination site lysine were not identified. It was determined that the location of the lysine residue was not restricted to a single site, as evidenced by substrate ubiquitination at both the N- and C-terminus, as well as at an internal lysine (Figure 4d). Next, overlapping shortened sequences were randomly selected from the parent p53 substrate in an attempt to isolate the smallest portable degron. Preliminary findings in this study identified an eight amino acid sequence, starting with the internal lysine residue, to be the most strongly ubiquitinated (Figure 4c, lane 5). As the p53-MDM2 interaction is currently the most desirable therapeutic target, a portable degron that could be incorporated into a reporter that can specifically evaluate Mdm2 (and the human homolog Hdm2) activity in patient samples are highly desirable [55].

In conclusion, the work presented here is the first step in the development of a novel reporter for proteasome activity using a portable degron as the targeting motif to recruit the reporter to the proteasome. The relatively rapid ubiquitination kinetics of degrons based on Bonger and iNOS demonstrate potential degrons that can be incorporated into a single cell proteasome reporter. Further work is required to increase peptide resistance to intracellular peptidases; however this analysis is a good preliminary examination into known degrons that could ultimately be incorporated into a proteasome reporter that is compatible with single cells and highly quantitative techniques such as capillary electrophoresis.

## Supporting Information

File S1 **Text S1, Figure S1–S4, Table S1.**
(DOCX)
