# Supplementary material for: A Comparative Analysis of the Ubiquitination Kinetics of Multiple Degrons to Identify an Ideal Targeting Sequence for a Proteasome Reporter
Source: PLoS One. 2013 Oct 29;8(10):e78082. doi: 10.1371/journal.pone.0078082 (PMC3812159; doi:10.1371/journal.pone.0078082)
Supplement: File S1 — Text S1, Figure S1–S4, Table S1. (DOCX) [file pone.0078082.s001.docx]

**Text S1: Kinetic Model Equations and Parameter Estimation**

The model developed in this paper uses first order reaction kinetics to estimate first order rate constants for ubiquitination and degradation of degron-based substrates. Due to the inclusion of ubiquitin aldehyde to inhibit deubiquitinating enzymes, substrate deubiquitination was neglected. Additionally, the proposed model ignores substrate polyubiquitination in favor of multi-monoubiquitination due to the inclusion of methylated ubiquitin, which cannot form polyubiquitin chains due to reductive methylation of lysine residues. This assumption was further validated by the failed regression of a second model proposing substrate polyubiquitination instead of multi-monoubiquitination (data not shown). The concentration of unbound substrate (C_1_) as well as mono- (C_2_), di- (C_3_), tri-(C_4_), and tetra-ubiquitinated (C_5_) substrates are governed by the following equations:

$\frac{dC_{1}}{dt}=-\left( k_{1}+k_{2}+k_{3}+k_{4}+k_{5} \right)C_{1}$ (Eq. S1)

$\frac{dC_{2}}{dt}=k_{1}C_{1}-k_{6}C_{2}$ (Eq. S2)

$\frac{dC_{3}}{dt}=k_{2}C_{1}-k_{7}C_{3}$ (Eq. S3)

$\frac{dC_{4}}{dt}=k_{3}C_{1}-k_{8}C_{4}$ (Eq. S4)

$\frac{dC_{5}}{dt}=k_{4}C_{1}-k_{9}C_{5}$ (Eq. S5)

With the following boundary conditions:

$@t=0, C_{1}=C_{0}, C_{2}=C_{3}=C_{4}=C_{5}=0$ (Eq. S6)

where C_0_ is the initial concentration of parent peptide on the 4.2 μg scale (concentration varied due to the different molecular weights of each peptide substrate but normally ranged from 12-14 µM). A degradation term (C_6_) could also be calculated based on the degradation rate constants k_5_-k_9_; however it was not calculated since degraded substrate could not be measured using the ubiquitin pull down assay. The experimental concentration values were determined by:

$C_{i}=C_{0}\frac{I_{exp}}{I_{parent}}$ (Eq. S7)

where C_i_ is the experimental concentration for a sample at a given time, I_exp_ is the intensity of the sample band determined by ImageJ and I_parent_ is the intensity of the unreacted parent peptide (included in every gel).

The MCMC algorithm determined the rate constants by successively minimizing the difference between the calculated concentration (C_2_-C_5_) and the experimental concentration (C_data_) using a cumulative sum of the squared deviation (cSSD):

$SSD= \sum_{i=1}^{5} \left( C_{data}-C_{model} \right)_{i}^{2}$ (Eq. S8)

$cSSD=\sum_{j=1}^{4} {SSD}_{j}$ (Eq. S9)

where C_data_ is the concentration measured at each time point for each ubiquitinated species and C_model_ is the concentration calculated using the above equations. In Eq. S8 the sum of the squared deviation (SSD) is calculated for a single ubiquitinated species (e.g. C_2_) across five time points (10, 30, 60, 120, 240 minutes). The SSD for each ubiquitinated species (C_2_ to C_5_) is then summed in Eq. S9 to generate the cSSD. An MCMC algorithm was written in MATLAB to perturb the system of ODEs until the cSSD reached a minimum value (usually a value in between 1-5). This value is depicted in Table 2 along with the rate constants calculated for each of the nine degron-based substrates. Finally, to assess goodness of fit between experimental data and model predictions correlation, coefficients were calculated for MonoUb~peptide, DiUb~peptide, TriUb~peptide, and TetraUb~peptide for all nine degron-based substrates using Eq. S10:

$r_{xy}=\frac{n\sum x_{i}y_{i}-\sum x_{i}\sum y_{i}}{\sqrt{\left[ n\sum x_{i}^{2}-\left( \sum x_{i} \right)^{2} \right]\left[ n\sum y_{i}^{2}-\left( \sum y_{i} \right)^{2} \right]}}$ (Eq. S10)

**Figure S1: Quantification of substrate ubiquitination.**


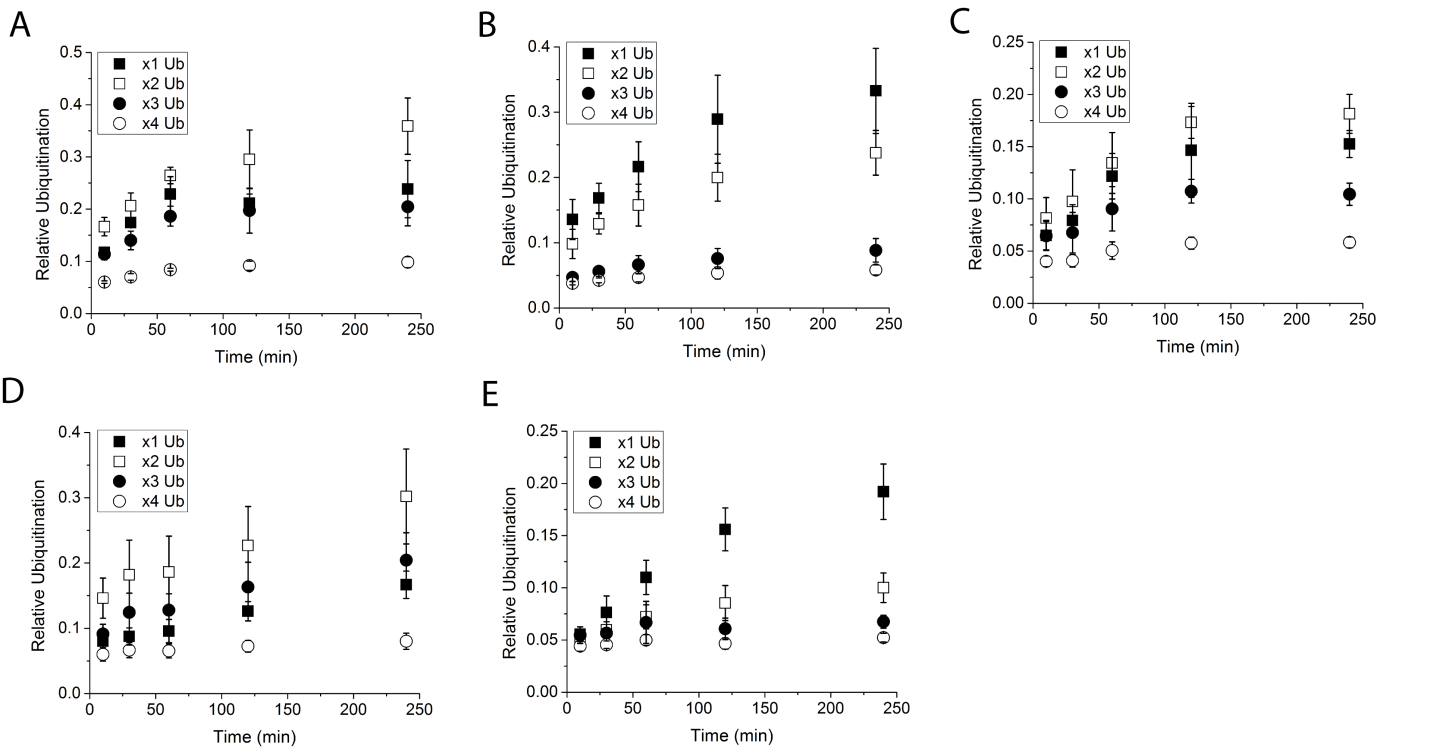


The remaining five members of the substrate library were evaluated with the ubiquitin pull down assay (with TUBES) and then visualized by the fluorescein tag. Relative ubiquitination values were calculated relative to unmodified parent using ImageJ as described in the methods. Five substrates based on degrons from (A) TAZ, (B) IFNAR1, (C) HIF-1-α, (D) Cyclin-D1, and (E) SRC3 were analyzed. Substrate sequences are included in Table 1. Mono-, di-, tri-, and tetra-ubiquitin conjugated substrates are labeled according to the legend. The data included is representative of three independent experiments.

**Figure S2: Degron-based substrate polyubiquitination**

**
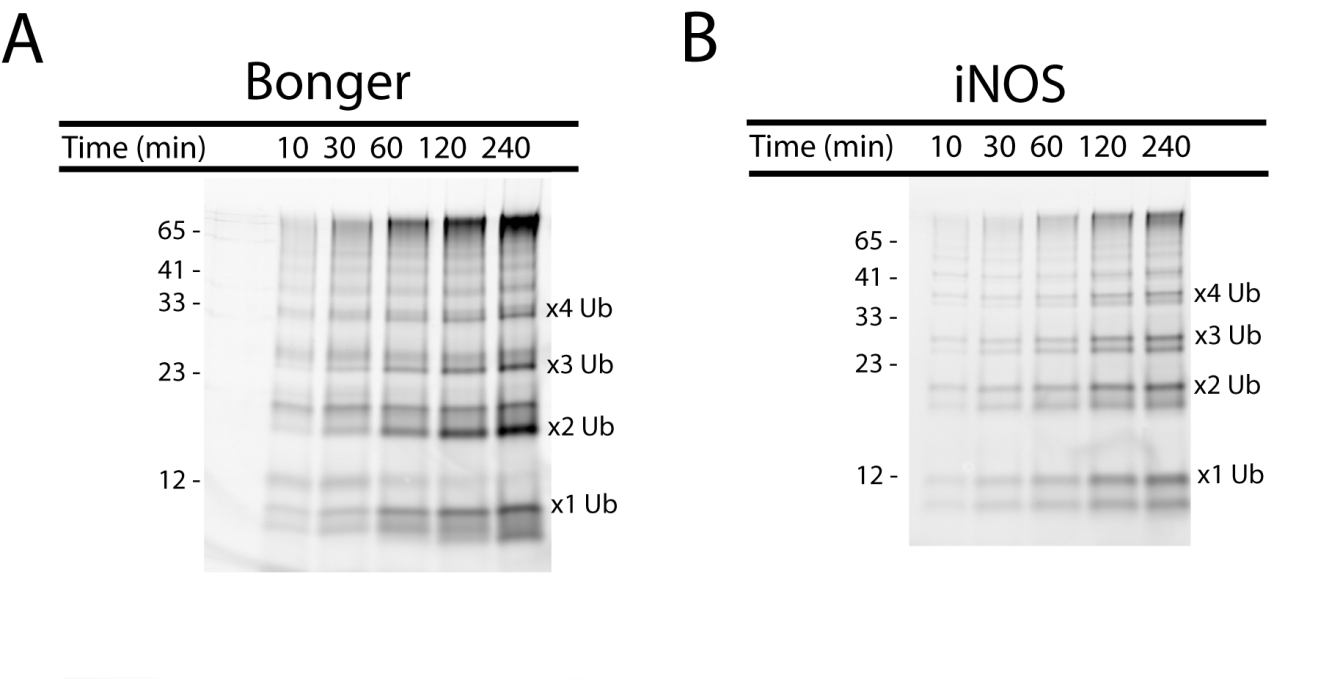
**

The ubiquitin pull down assay was performed for two model degron-based substrates, Bonger (A) and iNOS (B), in the presence of exogeneous ubiquitin (Ub) to demonstrate the higher molecular weight bands observed in the presence of MeUb migrate at the same location on the gel. The bands for mono- (x1), di- (x2), tri- (x3), and tetra- (x4) are indicated on the gel as well as the relative molecular weight as determined by a fluorescent protein standard as denoted to the left of the gel. Samples were isolated as described in the methods to separate ubiquitinated peptide and then the fluorescien tag was visualized by a Typhoon Imager.

**Figure S3: Proposed degron-based substrate polyubiquitination model.**


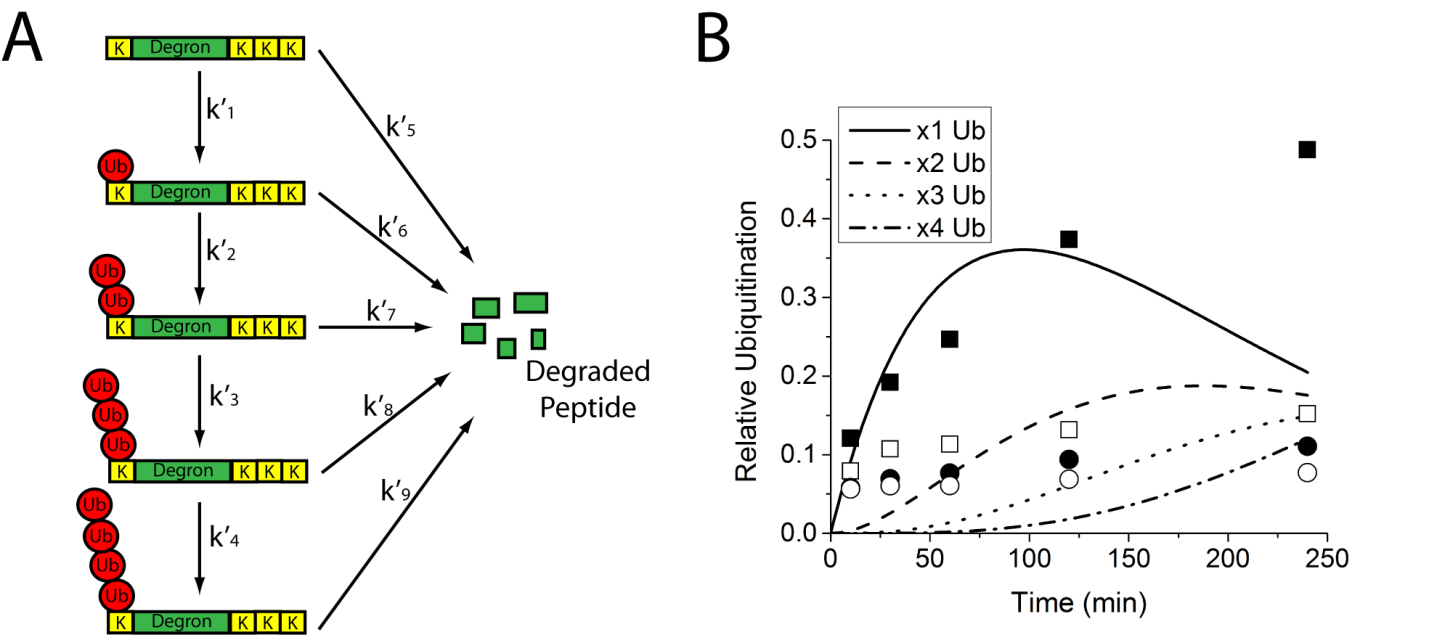


(A) In this model the generation of multi-ubiquitinated species comes only from the sequential addition of ubiquitins to the single, N-terminal lysine residue, forming a polyubiquitin chain, with each ubiquitin addition being governed by a corresponding rate constant (k’_1_-k’_4_). The model also addresses both unmodified and ubiquitin-conjugated substrate degradation (rate constants k’_5_-k’_9_). (B) The polyubiquitin model was unsuccessfully fit to the data set for the Bonger-based substrate (Figure 2d). The number of ubiquitin species conjugated to the substrate are denoted by filled squares (x1 Ub), empty squares (x2 Ub), filled circles (x3 Ub), and empty circles (x4 Ub). The cSSD for this fit was 23.360.

**Figure S4: Kinetic analysis of substrate ubiquitination to determine first order rate constants.**

**
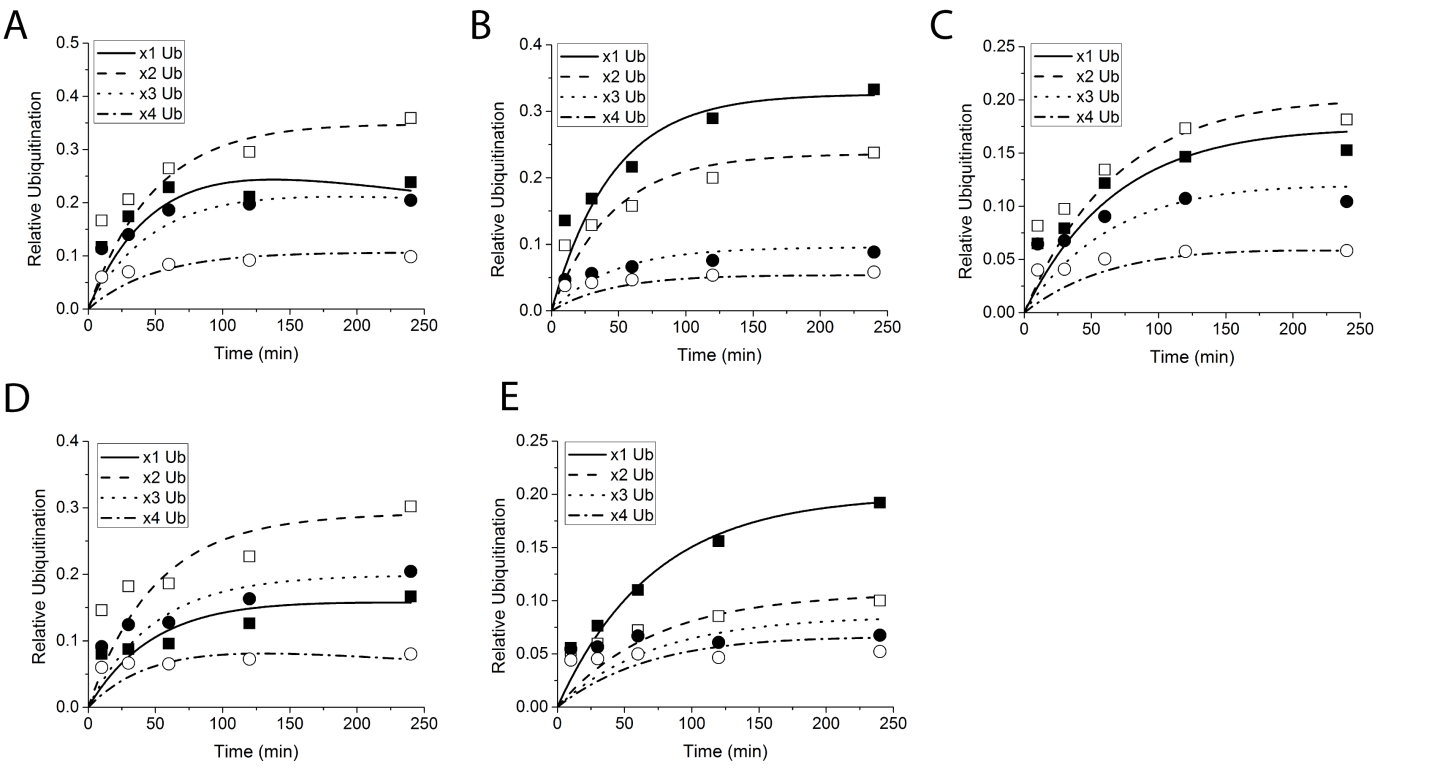
**

Rate constants correspond to Equations S1-S6 representing substrate ubiquitination and degradation. Model output for the remaining five substrates based on degrons from TAZ (A), IFNAR1 (B), HIF-1-α (C), Cyclin-D1 (D), and SRC3 (E) demonstrated a good fit between model prediction and observed data. Model output is depicted as relative ubiquitination (C_i_/C_o_) to allow for comparison across substrates. Multiple ubiquitins conjugated to a substrate are denoted by filled squares (x1 Ub), empty squares (x2 Ub), filled circles (x3 Ub), and empty circles (x4 Ub).

**Table S1: Correlation coefficients to measure the goodness of fit between the model predictions and experimental data.**

|  | **Correlation Coefficients (r_xy_)** | | | |
| --- | --- | --- | --- | --- |
|  | **Mono-Ub** | **Di-Ub** | **Tri-Ub** | **Tetra-Ub** |
| **Bonger** | 0.958 | 0.952 | 0.962 | 0.903 |
| **p53** | 0.932 | 0.842 | 0.849 | 0.753 |
| **iNOS** | 0.985 | 0.995 | 0.992 | 0.991 |
| **IFNAR1** | 0.960 | 0.963 | 0.967 | 0.964 |
| **TAZ** | 0.931 | 0.953 | 0.986 | 0.964 |
| **Cyclin D1** | 0.871 | 0.878 | 0.796 | 0.917 |
| **SRC3** | 0.990 | 0.987 | 0.795 | 0.791 |
| **HIF-1α** | 0.985 | 0.991 | 0.970 | 0.969 |
| **β-Catenin** | 0.971 | 0.962 | 0.979 | 0.902 |

Correlation coefficients (r_xy_) were calculated by Equation S10, comparing the five experimental data points to the corresponding time points from the kinetic model. As per convention, the correlation coefficient ranges between -1 and 1 with a positive value approaching 1 corresponding to a very good fit between model and data.
